# Unwanted experiences and support among men and trans individuals in the sex industry in Bangkok, Thailand

**DOI:** 10.3389/fsoc.2026.1738869

**Published:** 2026-02-23

**Authors:** Christina Duval, Shyla Bakshi, Celeste McGee, Glenn Miles, Jarrett Davis, Annina Janke, Madison Moore, Malinee Phosae, Madeline Stenersen

**Affiliations:** 1 Department of Psychology, Saint Louis University, St. Louis, MO, United States; 2 Dton Naam, Bangkok, Thailand; 3 Up! International, Berne, Switzerland

**Keywords:** gender-diverse, help-seeking, service-barriers, stigma, vulnerability

## Abstract

Male and transgender sex workers in Thailand experience high rates of violence, social stigma, economic marginalization, as well as limited access to legal protections and support services. However, this population remains significantly understudied in trafficking and sex work literature and often lacks adequate support services. As such, through quantitative surveys with 100 sex workers assigned male at birth (73% transgender/third gender, 26% cisgender) in Bangkok, this research explores their experiences of harm, knowledge of support services, and barriers to accessing assistance. Over half the participants (57%) reported experiencing at least one harmful event, most commonly emotional harm, stigmatization, and robbery. Despite these experiences, formal reporting was limited, with only 16% contacting police and 5% approaching NGOs, while most (37%) confided in friends or family. Only 20% of participants had ever accessed support resources. Key barriers to help-seeking included fear of stigma, concerns about judgment related to sexuality, and masculinity norms. Notably, transgender individuals showed greater willingness than cisgender men to report physical abuse to police. Findings highlight the distinct needs of gender-diverse sex workers and suggest targeted interventions to improve service accessibility, including anonymous reporting options, legal support, and culturally sensitive approaches that address both practical concerns and identity-based stigma.

## Introduction

Male and transgender individuals working in Thailand’s commercial sex industries face systematic vulnerabilities that remain largely undocumented and unaddressed. These include high rates of physical and sexual violence, pervasive stigma, and legal marginalization, with limited access to support services (Decker et al., 2022; Jirattikorn and Tangmunkongvorakul, 2023). Despite these known barriers, existing research and intervention efforts have primarily focused on cisgender women, leaving male and transgender individuals without adequate protection or support systems. This study addresses this critical gap by examining the experiences of 100 male and trans/third gender individuals working in Bangkok’s commercial sex industry, exploring their experiences of harm, knowledge of support systems, and barriers to accessing assistance.

### Theoretical framework: intersectionality and stigma

From a sociological perspective, the experiences of male and transgender sex workers in Thailand exemplify the intersection of multiple systems of marginalization including economic precarity, gender non-conformity, and occupational stigma. These intersecting vulnerabilities create what scholars term “fundamental causes” of health and social inequalities where stigmatized identities compound to limit access to resources and protective factors (Link and Phelan, 2001). Recent intersectional research emphasizes how interpersonal and structural forms of discrimination have compounding impacts on marginalized populations, particularly those navigating multiple stigmatized identities.

### Migration, economic Precarity, and entry into sex work

The prevalence of Thailand’s sex industry attracts sex workers and tourists internationally (Ocha, 2020). People often first migrate from nearby rural areas (Weitzer, 2021) or countries with economic turmoil, like Myanmar (Jirattikorn and Tangmunkongvorakul, 2023) to urban economic centers for personal and familial economic opportunities. As Weitzer (2023) documents in his comprehensive analysis of Thailand’s sex tourism industry, the experiences of sex workers vary considerably by gender, nationality, and work venue, yet male and transgender sex workers remain systematically understudied.

After working in the conventional job market, many may experience unsustainable wages or a lack of opportunities. For gender-diverse populations, minimum wages are compounded with discrimination in hiring practices based on their sexual orientation and gender (UNDP, 2014, as cited by Ocha, 2020). As a result, migrants and gender-diverse populations frequently seek economic relief by working in the sex industry through venues including karaoke bars, massage parlours, dance clubs, and go-go bars (Singh and Hart, 2007) or through online sexual services (Miles et al., 2024).

### Masculinity, gender identity and compound stigma

For cisgender male sex workers who service male clients, stigma operates through what Connell (1995) terms “hegemonic masculinity” meaning the cultural idealized form of masculine character that systematically subordinates both women and non-hegemonic masculinities. Recent ethnographic work reveals how heterosexually-identified male sex workers often perform “exaggerated masculinity” to negotiate tensions between their marginalized social positions and dominant masculine ideals (Özbay, 2010; Jirattikorn et al., 2022). This performance can have significant psychological and emotional consequences as men struggle to perform these ideals while maintaining their own sense of self.

Sex workers also endure several threats to their mental and physical wellbeing, including risks of HIV/AIDS, trafficking, and abuse (Singh and Hart, 2007). The risk of these issues was exacerbated by the COVID-19 pandemic, as sex workers were reported to be one of the ‘most vulnerable’ groups in terms of accessing support services due to the criminalization of their work (Janyam et al., 2020). These material vulnerabilities intersect with pervasive cultural stigma about sex work in Southeast Asian contexts, leading to public shame and social exclusion (Jirattikorn and Tangmunkongvorakul, 2023; McCamish, 2002; McKenzie et al., 2021; Weitzer, 2021).

### Support services: navigating between police and NGOs

In the evaluation of the support services offered to sex workers in Thailand, one primary outlet consists of the police. As sex work is illegal in Thailand, sex workers often avoid aid from the police out of fear of being arrested (Decker et al., 2022), but their prevalence throughout the country makes them also a resource for social services (Tenni et al., 2015). Researchers have also shown increased efforts among police to engage in positive outreach with sex workers, as demonstrated through their partnership with social services organizations, such as the Sex Workers in Network Group (SWING), in providing STI and HIV prevention and treatment without attempts to arrest (Tenni et al., 2015). Nevertheless, the persistence of stigma continues to force sex workers to make difficult decisions about disclosure, help-seeking, navigating complex calculations of risk versus potential support (Armstrong, 2022).

While non-governmental organizations (NGOs) attempt to provide legal, healthcare, and educational resources to sex workers (Barmania, 2013), they remain significantly underutilized compared to police services. In Miles et al. study that evaluated Thai sex workers’ reporting of abusive and exploitative experiences, only 11% of trans/third-gender participants chose to notify an NGO about their experience, whereas 19% chose to notify the police (2024). This underutilization reflects broader structural barriers: NGOs sometimes conflate consensual sex work with trafficking, leading to “anti-trafficking raids” that paradoxically harm the workers they aim to help (Singh and Hart, 2007).

### The current study

By applying an intersectional lens to understand how gender identity, occupational stigma, and masculinity norms shape help-seeking behavior among 100 male and transgender sex workers in Bangkok, this research aims to inform the development of more inclusive and effective support services. Through quantitative surveys examining experiences of harm, knowledge of support services, and barriers to accessing assistance, this study contributes to the growing body of sociological literature on how multiply-marginalized populations navigate systems of support and surveillance within contexts of structural inequality. By comprehensively understanding gender-diverse sex workers’ motivation to report harm and sexual exploitation, we can learn about what resources are specifically impactful and needed to assist them.

## Materials and methods

### Participants

Survey responses were collected from 100 sex workers who were assigned male at birth. All participants were over the age of 18 and were currently working in the sex industry in Bangkok, Thailand.

Data collection was led by the team at Dton Naam Foundation, a local service provider for LGBTQ+ sex workers located in Bangkok, Thailand, with the goal of informing service access and needs. Participants were recruited using a combination of purposive and snowball sampling methods. Initial participants were identified through Dton Naam’s existing outreach networks, with subsequent recruitment occurring through peer referral chains. This approach was necessary given the hidden nature of the population and the illegal status of sex work in Thailand.

Data was collected by a team of case workers employed Dton Naam, who conducted the interviews either in the field or at a nearby cafe. As a local service provider with established trust in the LGBTQ+ sex worker community, Dton Naam’s involvement facilitated access to this hard-to-reach population while providing a safe and familiar context for disclosure. The foundation’s reputation for non-judgmental support and their ongoing presence in Bangkok’s sex work venues enabled recruitment of participants who might otherwise avoid research participation.

Locations were chosen based on what made the participants feel the most secure. The surveys were completed digitally on either a phone or a tablet and included an informed consent process followed by a survey of experiences. No participants dropped out of participation following informed consent. The case workers stayed with the participant while they completed the survey to answer question and help with reading difficulties. Ethical approval for secondary analysis of this survey data was sought and obtained by the institutional review board at Saint Louis University.

### Measures

Participants responded to various questions created by the NGO and research team, including questions regarding their life experiences. The current study focuses on questions related to unwanted/traumatic experiences reported by participants, their knowledge of, and ability to access support services, and their experiences/hesitations with support services available to them. All questions were quantitative with the option for the participant to add an ‘other’ response and specify their answer. Surveys were administered in Thai to ensure linguistic accessibility. Responses were translated into English by bilingual research team members, with back-translation conducted on a subset of responses to ensure accuracy. Any discrepancies were resolved through team discussion. Specific questions are outlined in the Results section.

### Statistical analysis

Analyses included descriptive statistics (frequencies and percentages) for participant responses. Chi-square tests of independence were conducted to examine associations between gender identity (transgender vs. cisgender) and key outcome variables. Given the small cell sizes for some gender categories, Fisher’s exact test was used where appropriate. Statistical significance was set at *p* < 0.05.

## Results

### Participant demographics

Demographic items asked participants to identify their age, nationality, ethnicity, and gender. Participant ages ranged from 19 to over 50, with the majority of participants being under 35 years old. Participants primarily were born in Thailand (*n* = 81; 81%), with the other 29% (*n* = 29) hailing from Cambodia (*n* = 4; 4%), Laos (*n* = 11; 11%), or Myanmar (*n* = 4; 4%). However, only 22% (*n* = 22) of the sample were born in Bangkok, indicating that 78% (*n* = 78) of the participants were migrants to the city. Regarding ethnicity, 42% (*n* = 42) of the sample self-identified as Thai from Isaan backgrounds, 32% (*n* = 32) identified as ethnic minorities, and the remaining 26% (*n* = 26) identified as a range of ethnicities. The sample included a diverse range of genders, including third gender (*n* = 46; 46%), transgender women (*n* = 26; 26%), men who have sex with men (*n* = 19; 19%), gay men (*n* = 7; 7%), and ladyboy (*n* = 1; 1%). One client chose not to disclose their gender. Due to the varied sample and relatively small sample sizes for some of the groups, two broader categories were created for further analysis: ‘transgender,’ which included third gender, transgender women, and ladyboy (*n* = 73; 73%), and ‘cisgender,’ which included men who have sex with men and gay men (*n* = 26; 26%).

### Experiences of unwanted events

**Figure 1:**
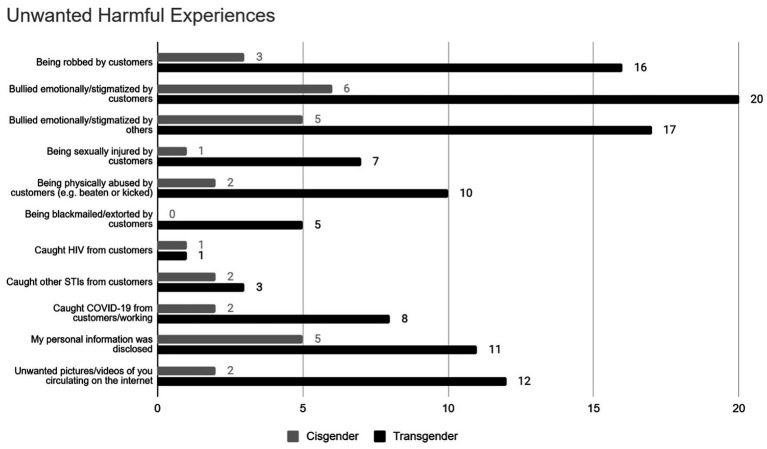
Unwanted harmful experiences reported by participants based on gender.

Respondents were presented with a list of harmful events and asked to select any they had experienced in the sex industry (full options displayed in Figure 1). A little over half of the participants (*n* = 57; 57%) reported having experienced at least one type of event, and a little over a quarter (*n* = 32; 32%) reported having experienced two or more events. The most commonly experienced events were emotionally harmful or stigmatising behaviours from customers (*n* = 27; 27%), emotionally harmful or stigmatising behavior from others (*n* = 23; 23%), and being robbed by customers (*n* = 19; 19%). Additionally, a fifth (*n* = 21; 21%) of participants reported having been either physically abused or sexually injured by customers, and a third (*n* = 31; 31%) reported having been forced to engage in unwanted sexual activity with a client. A chi-square analysis revealed no significant differences between experiences of harmful events based on gender (all *p*-values > 0.05).

### Accessing support and resources

Respondents who had experienced at least one unwanted event were then asked if, in response, what actions, if any, they had taken. While 37% (*n* = 37) talked with a friend or family member, 18% (*n* = 18) of participants reported taking no action at all. Only 21% (*n* = 21) reported that they reached out for additional support, with 16% (*n* = 16) talking with the police and 5% (*n* = 5) talking with an NGO.

Participants were also asked if they knew where to go if they did want to report an event that had happened. The majority of participants indicated that they would go to their local police (*n* = 72, 72%), followed by an NGO (*n* = 12, 12%), their place of work (*n* = 5, 5%), and the 191-emergency hotline (*n* = 3, 3%). Additionally, 14% (*n* = 14) of participants responded that they did not know where to go to make a report. A chi-square analysis revealed no gender differences in reporting history or preferences.

### Reporting to the police

Respondents were to review the previously presented experiences (outlined in Figure 1) and indicate whether they would be likely to contact the police for help after experiencing each. More than half of the participants reported that they would contact the police if they were robbed (*n* = 58; 58%) or physically abused (*n* = 63; 63%) by customers. On the other hand, only 20% (*n* = 20) would contact the police after being sexually assaulted by customers. Participants could select multiple options when indicating accommodations that would increase their likelihood of reporting. Participants primarily preferred being able to remain anonymous (*n* = 47, 47%) or having legal (*n* = 40, 40%) or NGO (*n* = 43, 43%) assistance when making the report.

A chi-square analysis revealed one significant difference between gender categories: transgender individuals are more likely to report physical abuse to the police than cisgender participants (*x* = 4.3, *p* < 0.05).

### Working with NGOs

Only a small minority of respondents had ever received resources from (*n* = 20; 20%) or disclosed trading sex to (*n* = 28; 28%) an NGO. The participants who had made contact with an NGO were primarily referred by outreach workers (*n* = 15; 75%), although participants on average had 2 different referral sources. These participants reported overwhelmingly positive experiences receiving services. All of the participants who had received resources from an NGO indicated that the organization was helpful and that the staff understood what they needed. Additionally, the majority reported that they met the criteria for services (*n* = 15; 75%), the staff cared about them (*n* = 17; 85%), and they were able to get the help they needed (*n* = 18; 90%).

Participants were also asked about factors that would make them more likely to report to an NGO. Participants could select multiple staff gender preferences, with 49% (*n* = 49) indicating that any gender would be fine. 46% (*n* = 46) of respondents indicated they would talk with a woman, 26% (*n* = 26) preferred a transgender person, and 16% (*n* = 16) would prefer a man. In terms of reporting methods, participants primarily preferred being able to report in person instead of online (*n* = 71, 71% vs. *n* = 30, 30%). Two respondents (2%) indicated they would not talk to an NGO under any circumstances. A chi-square analysis revealed no gender differences in NGO reporting history or preferences.

### Challenges in talking with service providers

**Figure 2:**
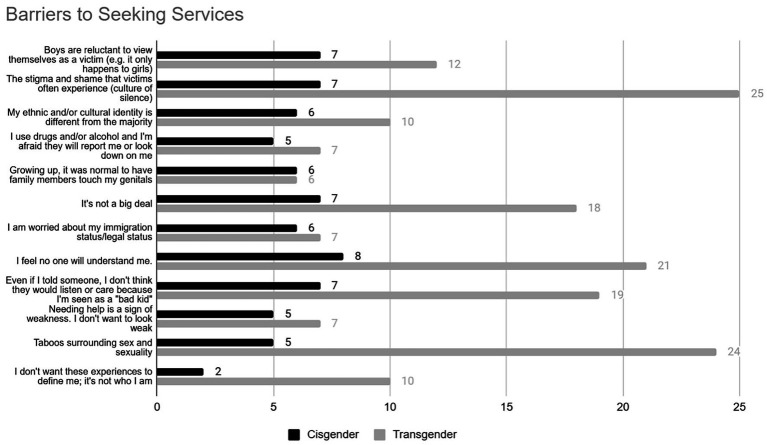
Barriers to seeking services reported by participants by gender.

Respondents were presented with a list of potential concerns and asked to select any they felt were barriers to speaking openly with service providers. Results revealed that participants had varied opinions, with no ‘challenge’ appearing overwhelmingly endorsed more than the others (Figure 2). Many of the most commonly selected challenges involved concerns about societal norms and stigma. Specifically, 32 participants (32%) endorsed fearing the ‘stigma and shame that victims often experience (culture of silence)’ and 29 participants (29%) endorsed concerns about ‘taboos surrounding sex and sexuality.’ A smaller subsection of participants was specifically concerned about stigma related to masculinity, with 19 participants (19%) endorsing ‘Boys are reluctant to view themselves as a victim (e.g. it only happens to girls)’ and 12 participants (12%) endorsing ‘Needing help is a sign of weakness. I don’t want to look weak.’

Some of the challenges focused more specifically on the implications of disclosing their experiences to providers. Specifically, participants were concerned about disclosing due to their ethnicity (*n* = 16; 16%; ‘My ethnic and/or cultural identity is different from the majority’), immigration status (*n* = 14; 14%; ‘I am worried about my immigration status/legal status’), and substance use (*n* = 12; 12%; ‘I use drugs and/or alcohol and I’m afraid they will report me or look down on me’). Many participants were also concerned about disclosing due to how they felt they were perceived, with 29 participants (29%) endorsing ‘I feel no one will understand me’ and 26 participants (26%) endorsing ‘Even if I told someone, I don’t think they would listen or care because I’m seen as a “bad kid.”’

Finally, a notable subsection of the participants indicated that they would not disclose to providers due to a lack of need or urgency. Specifically, 26 participants (26%) reported that they felt ‘it’s not a big deal.’

## Discussion

Our study sought to evaluate the factors that influence the reporting of harmful experiences among men and transgender individuals in Bangkok’s sex industry. The results provide empirical support for understanding how multiple, overlapping systems of marginalization—occupational stigma, gender identity, and masculinity norms—shape both experiences of harm and pathways to support. Consistent with Link and Phelan's (2001) conceptualization of stigma as operating across multiple levels, we found that 57% of participants endured unwanted, harmful events from customers, most commonly in the form of coerced sexual activity (31%) and emotional harm or stigmatization from clients (27%) and others (23%). These findings align with previous literature on the extensive prevalence of abuse within the Thai sex industry (Singh and Hart, 2007) and demonstrate how stigma manifests not only structurally through criminalization but also interpersonally through client and community interactions.

The results of the study also demonstrated that participants primarily seek support from friends and family compared to external sources. If they were to report an unwanted experience, the majority indicated they would go to the police (72%), especially if they were to be robbed or physically abused, followed by going to an NGO (12%). While certain research has demonstrated that sex workers often avoid aid from the police due to fear of arrest (Decker et al., 2022), our results indicate a willingness by participants to seek help in instances of violent crime, aside from cases of sexual abuse. Our results are consistent with past research that NGOs remain underutilized by sex workers compared to the police (Miles et al., 2024). In terms of the challenges sex workers faced in speaking with service providers, participants expressed concerns about stigma and shame, ethnic and personal identification, and a lack of understanding from others.

The findings illustrate the presence of gender disparities in the reporting of sexual exploitation that reflect broader patterns of hegemonic masculinity. Transgender sex workers were significantly more likely to report physical abuse to the police compared to cisgender individuals. These results replicate past research where cisgender males were less likely than trans/third-gender peers to disclose experiences of abuse and violence to others (Miles et al., 2024). These gendered patterns in help-seeking behavior can be understood through the lens of ‘hegemonic masculinity’ (Connell, 1995), where admitting victimization fundamentally challenges masculine identity. As documented in other contexts where heterosexually-identified men engage in same-sex sexual activity for economic survival (Özbay, 2010), our cisgender participants may experience a ‘double bind’—already navigating challenges to their masculine identity through their work, they may be particularly reluctant to further compromise this identity by positioning themselves as victims requiring assistance. This interpretation is supported by our finding that some participants explicitly identified masculinity-related barriers (e.g., ‘needing help is a sign of weakness’) as a barrier to speaking with service providers.

The results of our study reveal that the police play an important, but complicated, role in the support system of sex workers. Almost three-quarters of the participants indicated that, if they were to report a harmful experience, they would go to the police. Only 20% of participants reported alternate reporting locations. As such, the majority of the participants would only report their experiences to the police. Borrowing from the ‘realist’ approach used by Tenni et al. (2015), if participants are going to report to the police, local NGOs could benefit by cultivating relationships with their local police force. An example is the partnerships already being created in Bangkok by SWING (Tenni et al., 2015). NGO representatives could provide training and assistance to the police, instructing them on how to respond in a non-stigmatising or punitive manner. Additionally, police could provide referrals to local NGOs, thus increasing the population served and decreasing the burden on the police.

On the other hand, our results also indicate a potential gap in the impact of the police. When asked specifically about what kinds of events participants would report to the police, they primarily selected situations relating to physical and financial abuse. However, only 1 in 5 participants indicated that they would report sexual injury to the police. These results suggest that participants feel less comfortable coming to the police for concerns related to sex and sexuality, which could in part be motivated by the illegal status of sex work (Decker et al., 2022) and the continuing stigma against transgender female and male sex workers in Thailand. Thus, there is an important gap in services that NGOs could fill. It could prove beneficial for NGOs to work with community members to develop a pathway for reporting sexual harm and receiving support.

From a sociological perspective, our findings illustrate how structural inequality shapes individual agency in seeking support. The intersection of multiple marginalized identities—sex worker, gender minority, often migrant—creates what Crenshaw (1991) described as unique forms of discrimination that cannot be understood through single-axis frameworks. The fact that participants primarily relied on informal support networks (37% confided in friends/family) rather than formal services suggests that community-based, peer-led interventions might be more effective than traditional top-down approaches. This aligns with recent movements toward ‘community-centered’ rather than ‘rescue-oriented’ approaches in sex worker support services globally. The results implicate the importance of service providers facilitating gender-informed care when working with male and transgender sex workers in Bangkok’s sex industry, with police and NGOs initiating training and educational interventions that address the specific, intersecting barriers this population faces.

### Limitations and future directions

The current study represents community-collected research relevant to service providers and their participants but is not without its limitations. First, as the current study was quantitative, the full extent/context of unwanted and service provider experiences cannot be known. Future research may benefit from a qualitative/narrative structure that allows participants to elaborate on their experiences and produce more detailed responses. Second, participants, only a small percentage (20%) of participants reported receiving services from NGOs at any point. Though current results represent real fears and challenges with connecting with service providers, these responses may be different among a sample in which more participants had worked with an NGO previously or currently. Similarly, this sample represents a small portion of sex workers in Thailand and does not claim to be representative of all sex workers in the country. Third, the current study assessed lifetime prevalence of harmful experiences rather than frequency or recency. While this approach captures the breadth of experiences within this population, it cannot distinguish between isolated incidents and patterns of repeated harm. Future research should incorporate temporal and frequency measures to better understand the chronicity of these experiences and their differential impacts on help-seeking behavior. Fourth, though having local and known entities in case workers was beneficial in approaching participants, it is recognized that the use of snowball sampling and the case workers being known entities to participants may have influenced their answers throughout data collection. Similarly, asking participants to note engagement in illegal activities may have influenced responses. Finally, though the current study focuses on identifying unwanted experiences potentially relevant to service providers, future studies may benefit from an examination of strengths-based experiences and positive relationships with service providers as a way to also encourage relationships that support and appreciate participants in the sex industry and meet their needs.

## Conclusion

Despite significant mainstream attention to Thailand’s sex industry, this study represents one of the few systematic examinations of male and transgender sex workers’ experiences in collaboration with local service providers. Our findings reveal how intersecting systems of oppression—criminalization, gender-based stigma, and masculinity norms—create multiple barriers to support access. The significant proportion experiencing harmful events coupled with low formal service utilization underscores the urgent need for interventions that recognize and address these intersecting vulnerabilities. Collectively, these results represent multiple points of intervention, prevention, and potential contact for service providers looking to meet the needs of this population.

## Data Availability

The datasets presented in this article are not readily available because this dataset cannot be shared as it includes anonymized information but on a small population of individuals who may be easily identified through the sharing of even anonymized data. Requests to access the datasets should be directed to Madeline Stenersen, madeline.stenersen@health.slu.edu.
